# Telomere attrition and inflammatory load in major depressive disorder

**DOI:** 10.1192/j.eurpsy.2021.52

**Published:** 2021-08-13

**Authors:** A. Squassina

**Affiliations:** Department Of Biomedical Sciences, University of Cagliari, Cagliari, Italy

**Keywords:** aging, inflammation, telomere length, mood disorders

## Abstract

Patients with major depressive disorder (MDD) present features that suggest the involvement of accelerated aging, such as increased circulating inflammatory markers and shorter telomere length (TL). Here we measured T-lymphocytes TL with quantitative fluorescent in situ hybridization (Q-FISH) and plasma levels of inflammatory markers in a cohort of 37 patients with MDD and 36 non-psychiatric controls (C). TL was shorter in MDD compared to C (F=8.52, p=0.005). Patients with treatment resistant (TR) MDD showed higher levels of TNFα compared to non-TR (adjusted p= 0.034) and C (adjusted p=0.025), suggesting that treatment resistance might be associated with increased inflammation compared to non-TR.

**Disclosure:**

No significant relationships.

